# An Adaptive EEG Feature Extraction Method Based on Stacked Denoising Autoencoder for Mental Fatigue Connectivity

**DOI:** 10.1155/2021/3965385

**Published:** 2021-01-20

**Authors:** Zhongliang Yu, Lili Li, Wenwei Zhang, Hangyuan Lv, Yun Liu, Umair Khalique

**Affiliations:** ^1^College of New Materials and New Energies, Shenzhen Technology University, Shenzhen, Guangdong 518118, China; ^2^College of Heath Science and Environment Engineering, Shenzhen Technology University, Shenzhen, Guangdong 518118, China; ^3^School of Mechanical Engineering and Automation, Northeastern University, Shenyang 110819, China; ^4^College of Information, Liaoning University, Shenyang 110136, China; ^5^School of Mechanical Engineering, Xi'an Jiao Tong University, Xi'an 710049, China

## Abstract

Mental fatigue is a common psychobiological state elected by prolonged cognitive activities. Although, the performance and the disadvantage of the mental fatigue have been well known, its connectivity among the multiareas of the brain has not been thoroughly studied yet. This is important for the clarification of the mental fatigue mechanism. However, the common method of connectivity analysis based on EEG cannot get rid of the interference from strong noise. In this paper, an adaptive feature extraction model based on stacked denoising autoencoder has been proposed. The signal to noise ratio of the extracted feature has been analyzed. Compared with principal component analysis, the proposed method can significantly improve the signal to noise ratio and suppress the noise interference. The proposed method has been applied on the analysis of mental fatigue connectivity. The causal connectivity among the frontal, motor, parietal, and visual areas under the awake, fatigue, and sleep deprivation conditions has been analyzed, and different patterns of connectivity between conditions have been revealed. The connectivity direction under awake condition and sleep deprivation condition is opposite. Moreover, there is a complex and bidirectional connectivity relationship, from the anterior areas to the posterior areas and from the posterior areas to the anterior areas, under fatigue condition. These results imply that there are different brain patterns on the three conditions. This study provides an effective method for EEG analysis. It may be favorable to disclose the underlying mechanism of mental fatigue by connectivity analysis.

## 1. Introduction

Mental fatigue is a kind of key matter that threatens the traffic safety. It is very common during the daily life. Mental fatigue is defined to be the difficulties of initiating or maintaining initiative activity [1].The mental fatigue can lead to the decline of the alertness and vigor states accompanied by the tiredness, drowsiness, and difficulty in attention concentration. These manifestations are very dangerous for drivers after a long-time driving. Reports have shown that 16% traffic accidents are related to the mental fatigue of drivers [2]. Recently, many researchers have been devoted to mental fatigue effect [3–5], mental fatigue classification [6, 7], and fatigue countermeasures [8, 9]. In the view of biology, the mental fatigue is related to neuron energy reduction and glutamate transmission decrease [10]. Fatigue is also a comprehensive representation including both physiological and psychological elements [11]. The study about fatigue mechanism was elicited by auditory stimulus reports that mental fatigue is associated with the changes of brain activation on dorsal pathway [12]. It should be noted that the dorsal pathway has strong relationship with attention. The mental fatigue has also been proven to be related to the cognitive task, but not to be restricted to the performances of the stimulus-related brain areas [1]. Therefore, the exploration of the connectivity among multiareas of brain is favorable for illuminating the mechanism of mental fatigue.

Many kinds of measurements, such as face gestures [13] and neural signals [14], have been used to study mental fatigue. The electroencephalographic (EEG) as the direct and noninvasive measurement of the brain neuron activities has been regarded as one of the most applicable and reliable manifestation of the mental fatigue [15]. EEG primarily represents the excitement and inhibition of a mass of neurons' postsynaptic potentials, presenting a high temporal resolution [16]. EEG signals can be divided into delta (0.5 Hz-4 Hz), theta (4 Hz-7 Hz), alpha (8 Hz-13 Hz), beta (13 Hz-30 Hz), and gamma (30 Hz-80 Hz) activities, etc. Among these activities, the delta and theta activities have been proven to be related to fatigue condition [17]. These two activities have been used for the level of visual attention analysis [18], the mental fatigue evaluation [19, 20], and the fatigue prediction [21]. Methods, such as correlation analysis [22] and small-word network algorithm [23] are the common ways to analyze the brain connectivity. However, due to the attenuation of the brain structure, EEG manifests low signal-to-noise ratio (SNR) and space resolution. Noise may result in false connections between network nodes during connectivity analysis, thus blur the true connected relationship. Therefore, the effectiveness of these methods is limited. Brain source localization (BSL) algorithm [24] is a method to improve the space resolution and SNR through reconstructing the brain activity based on source. However, the low SNR of EEG still affects the solution of the ill inverse problem of BSL. The power spectrum density (PSD) of delta and theta activities has been widely employed for quantitative analysis of fatigue [25, 26]. However, the low SNR of EEG limits its application on connectivity analysis. Hence, a feature extraction algorithm is in urgent need for the analysis of mental fatigue. Murata and Uetake applied event-related potential and principal component analysis to extract the main fatigue feature from the blurred EEG [27]. Principal component analysis (PCA) is a common dimensionality reduction algorithm for improving SNR. It applies linear transformation to achieve a set of linearly independent components, thus to extract the principal feature. But, this algorithm may be limited during nonlinear EEG processing. The autoencoder is a novel nonlinear dimensionality reduction method. The stacked denoising autoencoder (SDAE) is a feedforward neural network which is consist of multiautoencoders. Its input signals are corrupted by noise. The hidden layer of SDAE that is restrained to be a narrow bottleneck can be considered as the reconstruction of original clean input signals. In this paper, in order to suppress the interference from noise, to decrease false connection between multibrain areas, and then to explore the underlying mechanism of mental fatigue, a novel model establishment method based on SDAE is proposed. This model is applied to extract features of mental fatigue under different kinds of fatigue conditions. The causal analysis of the extracted feature is applied for exploring the connectivity among multiareas of brain. This work provides a novel way to quantitatively analyze the mental states. It is also hopefully benefit to reveal the underlying synergistic effect between multibrain areas.

## 2. Dataset

**Figure 1:**
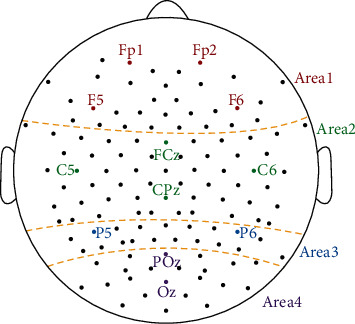
The electrode distribution of the mental fatigue experiment.

The experiment about mental fatigue includes fifteen subjects. The average age is 23.5 with a deviation of 1.37. All the subjects without any injured and diseased vision or diseases of central nervous system are from Northeastern University. All subjects give their informed consent for inclusion before they participate in the study. The study is conducted in accordance with the Declaration of Helsinki, and the protocol is approved by the institution's ethical review board of Northeastern University. The EEG data are recorded using a g.HIamp system (g.tec Inc., Austria) with a sampling rate of 1200 Hz from active 126 Ag/AgCl electrodes according to the 10-5 electrode location system [28]. The unilateral earlobe is chosen as the reference and the frontal position (Fpz) is adopted as the ground. To study the connectivity of the multibrain areas, the electrode distribution is subdivided into four areas, the frontal area (area 1), the motor area (area 2), the parietal area (area 3), and the visual area (area 4) according to the major function division of Brodmann area as shown in Figure 1.

The data are bandpass-filtered between 0.5 Hz and 100 Hz and notch-filtered from 48 Hz to 52 Hz to suppress noise. During the whole experiment process, all electrode impedances are kept below 30 k*Ω*.

Subjects are, respectively, seated in an armchair in a dark and electromagnetic shielding laboratory. The computer screen on the desk is about 1 meter away from the tip of subjects' noses. To suppress eyeball movement, subjects are instructed to focus on the screen center and to reduce the body movements. To record the EEG under awake condition, experiments are often carried out at about 9 am. The data collection on each subject lasts for about one minute. Afterwards, a consecutive P300 training section which continues for at least an hour is executed. Subjects are required to concentrate on the computer screen center and to type words from an English passage by P300 system. The line and row of P300 system lighten randomly. The subjects focus on the character that they should type. Then, about one minute EEG data are recorded as fatigue condition. After that, subjects need to keep awake for a whole night. They are not allowed to attend any entertainment activities. In the following day, about one minute EEG recording is collected at about 8 am on the same subjects as sleep deprivation condition.

## 3. Methodology

**Figure 2:**
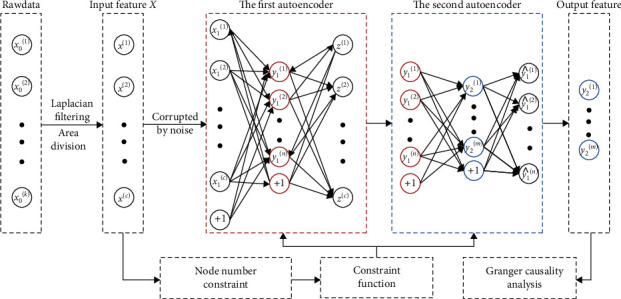
The flowchart of the EEG processing method.

The flowchart of the EEG processing method we applied is illustrated in Figure 2. Before data analysis, to reduce the influences of the volume conduction and to improve SNR and spatial resolution, the surface Laplacian algorithm has been applied on EEG recordings. It is formulated as Equation (1).

(1) $$\begin{array}{c} V_{C}=V_{CO}-\frac{1}{4}\left(V_{1}+V_{2}+V_{3}+V_{4}\right), \end{array}$$

where *V*_*C*_ is the signal after surface Laplacian filter. *V*_*CO*_ is the original signal. *V*_1_, *V*_2_, *V*_3_, and *V*_4_ are the signals around original signal. The locations of *V*_1_, *V*_2_, *V*_3_, and *V*_4_ are symmetrical in pairs. The center of symmetry is the location of *V*_*CO*_. The angle between adjacent two locations from *V*_1_, *V*_2_, *V*_3_, and *V*_4_ is 90 degrees.

### 3.1. The Stacked Denoising Autoencoder

The stacked autoencoder is an artificial neural network architecture, comprised of multiple autoencoders and trained by greedy layer wise training. Each autoencoder includes the middle layer, the output layer, and the input layer. The output of the middle layer acts as the input of the next autoencoder in the stacked autoencoder. The SDAE is the extension of the stacked autoencoder. The input signals of SDAE are corrupted by noise. To decode and recover the blurred original input EEG *X* = [*x*^(1)^, *x*^(2)^, ⋯, *x*^(*c*)^] from noise, a brief model of SDAE with two autoencoders is applied in this study. *c* is the channel number of input signal. The corrupted signals in the input layer is *X*_1_ = [*x*_1_^(1)^, *x*_1_^(2)^, ⋯, *x*_1_^(c)^]. These corrupted input signals are mapped to a hidden layer with *n* units by sigmoid function as Equation (2) [29].

(2) $$\begin{array}{c} Y_{1}={\text{f}}_{1,\theta }\left(X_{1}\right)=\text{s}\left({WX}_{1}+\text{b}\right), \end{array}$$

(3) $$\begin{array}{c} \text{s}\left(a\right)=\frac{1}{1+e^{-a}}, \end{array}$$

where *Y*_1_ is the signal on middle layer of the first autoencoder; *W*, *b*, and *f* are the weight matrices, bias, and activation function of encoder on the first autoencoder, respectively.

The weight and bias matrices are random assignment on the initialization stage. The uncorrupted input *Z* = [*z*^(1)^, *z*^(2)^, ⋯, *z*^(c)^], the estimation of *X*, can be reconstructed by the decoder of the first autoencoder as Equation (4).

(4) $$\begin{array}{c} Z=g_{2,\theta \prime }\left(Y_{1}\right)=\text{s}\left(W^{\prime }Y_{1}+{\text{b}}^{\prime }\right), \end{array}$$

where *W*′, *b*′, and *g* are the weight matrices, bias, and nonlinear function of decoder on the first autoencoder, respectively.

To minimize the average reconstruction error, the squared error loss function *L* is applied during the training of the structure parameters *θ* = (*W*, *b*) and *θ*′ = (*W*′, *b*′) of SDAE model. The optimization of these parameters is expressed in Equation (5).

(5) $$\begin{array}{c} \left\{\tilde{\theta },{\tilde{\theta }}^{\prime }\right\}=\underset{\theta }{\arg}\underset{\theta \prime }{\min}L\left(Z,{\text{f}}_{1}\left(X_{1}\right)\right)=\underset{\theta }{\arg}\underset{\theta \prime }{\min}\text{L}\left(f_{1}\left(X_{1}\right),g_{2,\theta \prime }\left(f_{1,\theta }\left(X_{1}\right)\right)\right). \end{array}$$

After the optimization, the first autoencoder has been established. The middle layer output of the first autoencoder is regarded as the input of the next autoencoder to further train the model of the second autoencoder. The output of the middle layer *Y*_2_ = [*y*_2_^(1)^, *y*_2_^(2)^, ⋯, *y*_2_^(*m*)^] of the second autoencoder has been considered as the deeply denoising feature extracted from the original input signals *X*.

### 3.2. Model Selection

In this study, to extract the EEG features with high signal to noise ratio under three conditions, the node numbers of middle layer on two autoencoders should be constrained. *n* and *m* are the node numbers of middle layer on first autoencoder and second autoencoder. For dimensionality reduction, *n* and *m* should be less than the number of nodes in their input layer. Then, the middle layer can be deemed as the dimensionality reduction of the input signals. The proposed constraints of *n* and *m* are formulated as Equation (6). The constraint function is formulated as Equation (7). A higher *c*_*f*_ indicating a better balance between model error and performance on feature extraction is a better selection.

(6) $$\begin{array}{c} \left\{\begin{array}{c} m\times n=U_{def},\\ m\leq n<C_{def},\\ 1\leq m<C_{com}, \end{array}\right. \end{array}$$

(7) $$\begin{array}{c} c_{f}=\max\left[\lambda e^{\left(-1/2c\left(\overset{c}{\underset{i=1}{\sum }}{\left\|x^{\left(i\right)}-z^{\left(i\right)}\right\|}^{2}+\overset{n}{\underset{i=1}{\sum }}{\left\|{y_{1}}^{\left(i\right)}-{{\overset{\wedge }{y}}_{1}}^{\left(i\right)}\right\|}^{2}\right)\right)}+\left(1-\lambda \right){\left(\overset{d}{\underset{j=1}{\sum }}c_{\text{STFT}}\left(j\right)\right)}^{3}\right], \end{array}$$

(8) $$\begin{array}{c} c_{\text{STFT}}\left(i\right)=\frac{\sum_{j=1}^{q}c_{\text{STFT}}\left(i,j\right)-\min\left(\sum_{j=1}^{q}c_{\text{STFT}}\left(i,j\right)\right)}{\max\left(\sum_{j=1}^{q}c_{\text{STFT}}\left(i,j\right)\right)-\min\left(\sum_{j=1}^{q}c_{\text{STFT}}\left(i,j\right)\right)}, \end{array}$$

where *U*_*def*_, *C*_*def*_, and *C*_*com*_ are a positive integers. *C*_*def*_ is smaller than the number of input channel. ${\hat{y}}_{1}=\left[{{\overset{\wedge }{y}}_{1}}^{\left(1\right)},{{\overset{\wedge }{y}}_{1}}^{\left(2\right)},\cdots ,{{\overset{\wedge }{y}}_{1}}^{\left(n\right)}\right]$ is the output of the second autoencoder. *c*_STFT_(*i*, *j*) is the short-time Fourier transform coefficients. *q* is the time sampling number. *d* is the concerned frequency sampling number.

**Figure 3:**
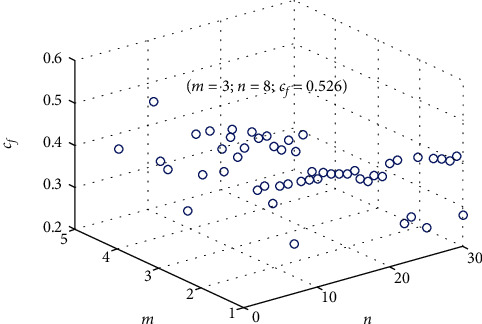
The results of *c*_*f*_ on the awake condition. *n* and *m* are the node numbers of middle layer on first autoencoder and second autoencoder, respectively.

In this study, let *U*_*def*_ = 30, *C*_*def*_ = 15, *C*_*com*_ = 5, and *λ* = 0.2. For the awake, fatigue, and sleep deprivation conditions, the main features of EEG are mainly under 20 Hz. Therefore, in this study, *d* is the frequency sampling number under 20 Hz. The signals from different areas are calculated by the SDAE model above, respectively. The average of output feature is applied as the extracted feature from the input layer. The results of *c*_*f*_ on the awake condition is illustrated in Figure 3. The pair of *m* and *n* obtaining higher *c*_*f*_ should be selected as the node numbers. In consideration of three conditions, the fine-tuned parameters for training the SDAE model of the experimental data are illustrated in Table 1.

**Figure 4:**
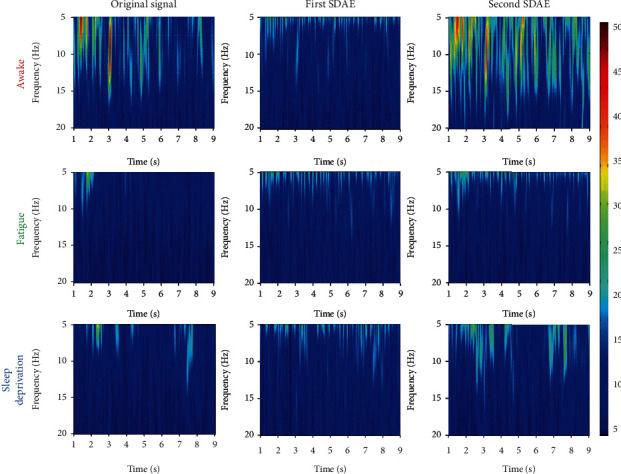
The time-frequency images of the average original signal and the features extracted by the first autoencoder and second autoencoder on area 4 during three conditions.

To study the extracted feature from SDAE model, the features extracted by the first and second autoencoders, and original signal have been analyzed by short-time Fourier transform. The time-frequency images of the average original signal, the feature extracted by the first autoencoder, and the feature extracted by the second autoencoder have been shown in Figure 4. Figure 4 indicates that the brain activities with high amplitude are highlighted by the second autoencoder.

The short-time Fourier transform coefficients of the extracted features and original signal have been normalized as Equation (8). The average *c*_STFT_ under 20 Hz is analyzed by *t*-test. Statistical results show that the features extracted by the second autoencoder obtains significant greater coefficients than the original signal (*P* < 0.001) and the features extracted by the first autoencoder (*P* < 0.001). Therefore, the output of the proposed model can significantly highlight EEG with high amplitude under 20 Hz.

**Figure 5:**
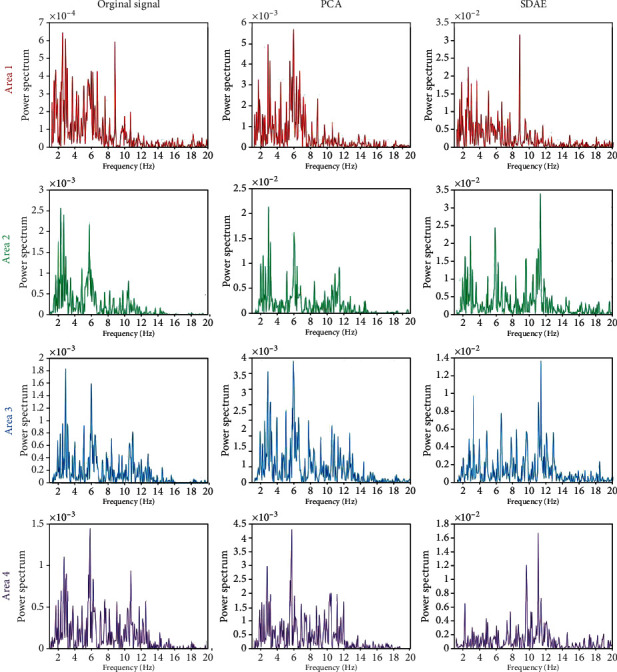
The power spectrum of the average original signal, the extracted feature by PCA, and the extracted feature by SDAE from four areas on awake condition.

**Figure 6:**
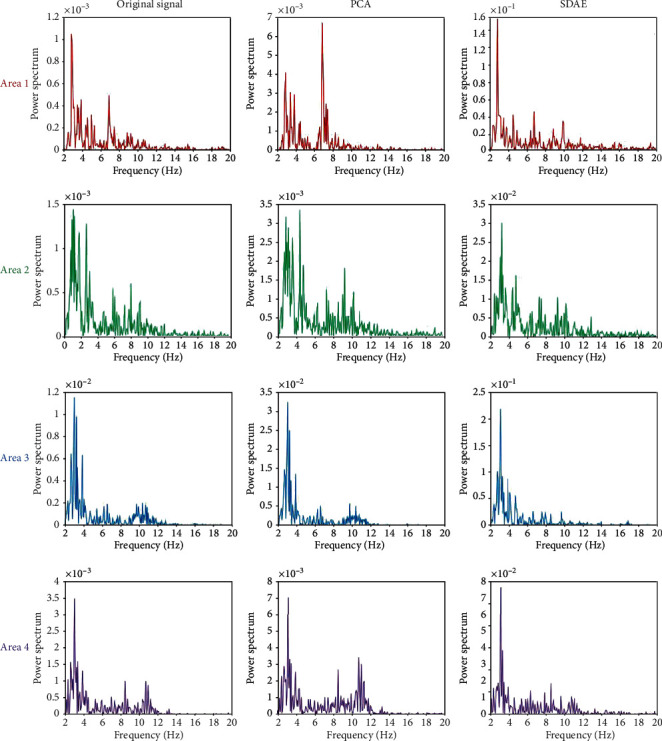
The power spectrum of average original signal, the extracted feature by PCA, and the extracted feature by SDAE from four areas on fatigue condition.

**Figure 7:**
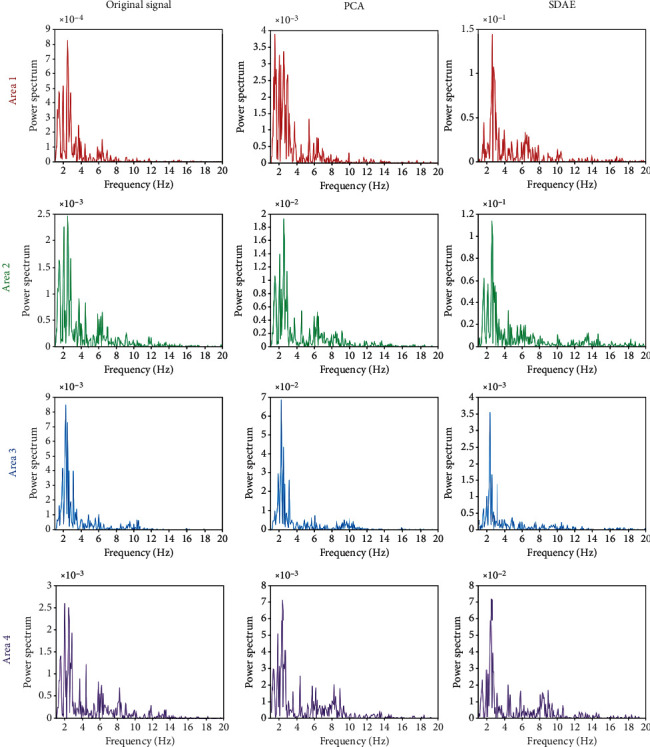
The power spectrum of average original signal, the extracted feature by PCA, and the extracted feature by SDAE from four areas on sleep deprivation condition.

To evaluate the performance of the proposed model selection method on EEG feature extraction, PCA algorithm has been applied for comparison.  Figure 5 illustrates the power spectrum of the average original signal across channel, the extracted feature by PCA, and the extracted feature by SDAE from four areas on awake condition. Figures 6 and 7 illustrate the fatigue condition and the sleep deprivation condition, respectively. Previous study indicates that alpha and beta frequencies dominate the common brain activities of human during the awake condition [30], and delta and theta activities have been proven to reflect mental fatigue. In Figure 5, under the awake condition, the original signal has been polluted by the low-frequency interference which is common in EEG. Alpha and beta frequencies are not obvious. The proposed model can extract the alpha frequency activity from the blurred signal and suppress the interferences from other frequencies, while PCA still remains these low-frequency interferences on the extracted feature. Under the fatigue and sleep deprivation conditions, the delta and theta activities dominate the brain activities with noticeable first primary frequency as shown in Figures 6 and 7. It is hypothesis that the first primary frequency contains important information about the brain activities. The ratio of the power on the first primary frequency and the average power (RPFA) on the concerned frequencies (awake: alpha and beta; mental about fatigue: delta and theta) is analyzed by *t*-test and illustrated in Table 2. A higher RPFA indicates a greater signal to noise ratio. Statistical results show that the proposed model obtains a significant greater RPFA than the original signal (*P* < 0.01) and PCA (*P* < 0.05). These results above indicate that the proposed model achieves a better performance compared with PCA in extracting feature from the blurred original EEG data, highlighting the first primary frequency and improving SNR.

### 3.3. Granger Causality Analysis

The features extracted by the proposed model are applied to the Granger causality analysis (GCA) to explore the connectivity among multibrain areas under the fatigue, awake, and sleep deprivation conditions. The GCA algorithm is a statistical method based on the forecast of time sequence. The causality relationship between sequences represents a better prediction accuracy on one time sequence with the prior knowledge of another time sequence. In order to explore the connectivity among multibrain areas, the multiple vector autoregressive model has been employed in this study [31]. *Y*_2_ = [*y*_2_^(1)^(*t*), *y*_2_^(2)^(*t*), ⋯*y*_2_^(*m*)^(*t*)] denotes the extracted feature. *m* is the vector number of *Y*_2_. The mutual prediction model is formulated as Equation (9).

(9) $$\begin{array}{c} y_{2}^{\left(1\right)}\left(t\right)=\overset{p}{\underset{j=1}{\sum }}C_{11,j}y_{2}^{\left(1\right)}\left(t-j\right)+\overset{p}{\underset{j=1}{\sum }}C_{12,j}y_{2}^{\left(2\right)}\left(t-j\right)+\cdots +\overset{p}{\underset{j=1}{\sum }}C_{1m,j}{\text{y}}_{2}^{\left(m\right)}\left(t-j\right)+\xi_{1}\left(t\right)\\ y_{2}^{\left(2\right)}\left(t\right)=\overset{p}{\underset{j=1}{\sum }}C_{21,j}y_{2}^{\left(1\right)}\left(t-j\right)+\overset{p}{\underset{j=1}{\sum }}C_{22,j}y_{2}^{\left(2\right)}\left(t-j\right)+\cdots +\overset{p}{\underset{j=1}{\sum }}C_{2m,j}{\text{y}}_{2}^{\left(m\right)}\left(t-j\right)+\xi_{2}\left(t\right)\\ \vdots \\ y_{2}^{\left(m\right)}\left(t\right)=\overset{p}{\underset{j=1}{\sum }}C_{m1,j}y_{2}^{\left(1\right)}\left(t-j\right)+\overset{p}{\underset{j=1}{\sum }}C_{m2,j}y_{2}^{\left(2\right)}\left(t-j\right)+\cdots +\overset{p}{\underset{j=1}{\sum }}C_{mm,j}y_{2}^{\left(m\right)}\left(t-j\right)+\xi_{m}\left(t\right), \end{array}$$

where *p* is the maximum number of lagged observations; *ζ*_1_, *ζ*_2_, and *ζ*_*m*_ are the prediction errors; *C* is the coefficient of the multiple vector autoregressive model.

The maximum number of lagged observations *p* is estimated by the ratio of the Akaike information criterion and the Bayesian information criterion. The noise covariance matrix is shown in Equation (10).

(10) $$\begin{array}{c} \Sigma =\left[\begin{array}{cccc} \mathrm{var}\left(\xi_{1}\right) & \mathrm{cov}\left(\xi_{1},\xi_{2}\right) & \cdots & \mathrm{cov}\left(\xi_{1},\xi_{m}\right)\\ \mathrm{cov}\left(\xi_{2},\xi_{1}\right) & \mathrm{var}\left(\xi_{2}\right)\; & \cdots & \mathrm{cov}\left(\xi_{2},\xi_{m}\right)\\ \vdots & \vdots & & \vdots \\ \mathrm{cov}\left(\xi_{m},\xi_{1}\right) & \mathrm{cov}\left(\xi_{m},\xi_{2}\right) & \cdots & \mathrm{var}\left(\xi_{m}\right) \end{array}\right]=\left[\begin{array}{cccc} \Sigma_{11} & \Sigma_{12} & \cdots & \Sigma_{1m}\\ \Sigma_{21} & \Sigma_{22} & \cdots & \Sigma_{2m}\\ \vdots & \vdots & \vdots & \vdots \\ \Sigma_{m1} & \Sigma_{\text{m}2} & \cdots & \Sigma_{mm} \end{array}\right]. \end{array}$$

The Granger causality from *y*_2_^(2)^(*t*) to *y*_2_^(1)^(*t*), conditioned on *y*_2_^(3)^(*t*) ⋯ *y*_2_^(*m*)^(*t*) is elaborated as Equation (11).

(11) $$\begin{array}{c} {F_{2\to 1}}_{\;|\;3\cdots m}=\ln\frac{\mathrm{var}\left(\xi_{1r}\right)}{\Sigma_{11}}, \end{array}$$

where var(*ζ*_1*r*_) is the component of the upper left corner in the noise covariance matrix of the restricted model omitted *y*_2_^(2)^(*t*).

## 4. Results

**Figure 8:**
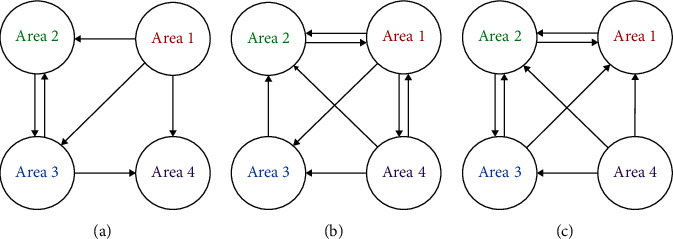
The causal connectivity of the features extracted by the proposed model under the three conditions: (a) the awake condition; (b) the fatigue condition; (c) the sleep deprivation condition. The connection between areas is significant (*P* < 0.01).

**Figure 9:**
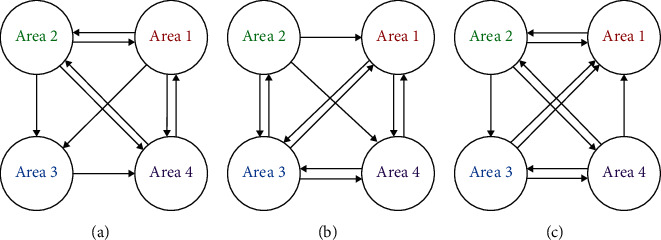
The causal connectivity of the features extracted by PCA under the three conditions: (a) the awake condition; (b) the fatigue condition; (c) the sleep deprivation condition. The connection between areas is significant (*P* < 0.01).

To study the connectivity between multibrain areas, the extracted features by the proposed model and PCA on the area 1, area 2, area 3, and area 4 have been analyzed by Granger causality analysis. The connectivity between areas is illustrated in Figures 8 and 9, respectively. In Figures 8 and 9, a, b, and c represent the awake condition, the fatigue condition, and the sleep deprivation condition, respectively. The connection between areas is significant (*P* < 0.01).

## 5. Discussion

The SDAE is a novel feature extraction method. In this study, the proposed model based on SDAE algorism has been applied on the analysis of EEG data about mental fatigue. Figures 5–7 indicate that the proposed model has an excellent performance on feature extraction of three conditions. It is should be noted that the concerned frequency range is different on three conditions. To study this, the results by short-time Fourier transform on the original signal and the features extracted by the first and second autoencoders are analyzed. It indicates that the proposed model is sensitive to brain activities with higher amplitude. Comparing with other conditions, the awake condition owns higher brain activities on mu and beta rhythms. The first autoenconder may focus on the contrast of light and shade. The second autoenconder may focus on amplitude difference. Therefore, in the awake condition, the information with high amplitude on mu and beta rhythms has been extracted and highlighted by the second autoencoder. Similarly, in the fatigue and sleep deprivation conditions, the information with high amplitude on delta and theta has been extracted and highlighted. Therefore, the model we proposed is an efficient and adaptive method on the analysis of EEG data about mental fatigue.

Figure 9 shows more bidirectional connections between areas than Figure 8. Most of the connection relationship in Figure 8 has been involved in Figure 9. These results have demonstrated the outstanding capability of the proposed model on extracting the main features from the blurred EEG, avoiding false connections and improving SNR compared with PCA. In Figure 8, the connectivity based on the features extracted by the proposed model under the awake condition presents a significant connection from the area 1 to its posterior areas in a vertical view. The connectivity under the fatigue condition reveals a complex trajectory, from the area 1 to its posterior areas and from posterior areas to the anterior areas. For the connectivity under the sleep deprivation condition, there is a causal flow from the area 4 to its anterior areas. There are different connected patterns on different brain mental states. On a paired connection relationship, the starting node contains important information that can be used to forecast the information of ending node. Therefore, the connection relationship may imply the process of information transmission on brain. The frontal area dominates the attention [32]. It is proved to be more activated with the increasing of the task complexity [33]. In this study, the results about EEG connectivity under the awake condition indicate that the area 1 plays an important role, and it may dominate brain activities. However, the awake condition does not contain any external mental concentrated task. Therefore, the awake condition may be not just an idling state, but an internal state requiring high concentrated attention. Dimitrakopoulos et al. indicate the information fluxion from the anterior areas to the posterior areas and the reverse under a one-hour simulated driving and a half-hour sustained attention task [34]. In this study, the complex bidirectional causal fluxion has been uncovered under the fatigue condition. To compare with the obvious unidirectional fluxion under the awake and sleep deprivation conditions, the complex connectivity between multibrain areas under the fatigue condition reveals that there may be a synergy or cross influence of multibrain areas after a long-time high attention-demanded task. Under the sleep deprivation condition, there is a causal flow from the area 4 to its anterior areas. Kar and Routray report that there are strong connections between electrodes on visual area during sleep deprivation [35]. Sleep deprivation is proved to slow the visual processing and to compromise the ability of visual stimuli processing [36]. Hereby, the connectivity of sleep deprivation condition indicates that the area 4 dominates the mental state with the visual processing suppression. This suppression may affect the other areas of the brain.

## 6. Conclusions

Fatigue is a common phenomenon during the period of performing cognitive task. In this study, to overcome the influence from noise and to study the underlying mechanism of fatigue, the model establishment method based on SDAE has been proposed. The proposed model has been applied to extract EEG features. The results have indicated that the proposed method can significantly improve SNR of the extracted feature. The causal connectivity of the extracted feature between multibrain areas under the awake condition, the fatigue condition, and the sleep deprivation condition has been studied. Different directions of causal flow have been revealed. The causal flow directions under the awake condition and the sleep deprivation condition are unidirectional but opposite. The connectivity under the fatigue condition exhibits the most complex trajectory between areas. It reveals a bidirectional causal fluxion, from the anterior areas to the posterior areas and from the posterior areas to the anterior areas. These results may reveal that different condition owns different underlying synergistic way between multibrain areas. This work provides a novel way to quantitatively analyze the mental states. It will be helpful to disclose the underlying mechanism of mental fatigue.

## Figures and Tables

**Table 1:** The parameter setting of the SDAE model.

Parameter name	Parameter value
Corrupted fraction probability	0.5
Learning rate	1
Minibatch size	200
Epoch number	8
Node number of the middle layer on the first autoencoder	8
Node number of the middle layer on the second autoencoder	3

**Table 2:** RPFA on the three conditions.

Condition	Area	Original data	PCA	SDAE
Awake	1	19.12	12.05	21.83
	2	9.97	9.30	13.27
	3	8.57	7.14	12.10
	4	9.51	6.83	12.51
Fatigue	1	8.72	8.97	11.45
	2	5.49	5.30	6.51
	3	10.75	12.17	11.86
	4	9.91	9.63	11.67
Sleep deprivation	1	8.73	6.38	8.75
	2	7.22	8.02	8.53
	3	25.84	30.80	31.64
	4	6.97	8.04	8.73
Average		10.90	10.39	13.24

## Data Availability

Please visit https://pan.baidu.com/s/1fuAc34TVkwnKouFqFwBQug. The access number is 11vp.

## References

[1] Ishii A., Tanaka M., Watanabe Y. (2014). Neural mechanisms of mental fatigue. *Reviews in the Neurosciences*.

[2] Wang H., Zhang C., Shi T., Wang F., Ma S. (2015). Real-time EEG-based detection of fatigue driving danger for accident prediction. *International Journal of Neural Systems*.

[3] Boksem M. A., Meijman T. F., Lorist M. M. (2005). Effects of mental fatigue on attention: an ERP study. *Cognitive Brain Research*.

[4] Tanaka M., Shigihara Y., Ishii A., Funakura M., Kanai E., Watanabe Y. (2012). Effect of mental fatigue on the central nervous system: an electroencephalography study. *Behavioral and Brain Functions*.

[5] Tanaka M., Ishii A., Watanabe Y. (2014). Neural effect of mental fatigue on physical fatigue: a magnetoencephalography study. *Brain Research*.

[6] Chai R., Tran Y., Naik G. R. Classification of EEG based-mental fatigue using principal component analysis and Bayesian neural network.

[7] Kai-Quan S., Chong-Jin O., Xiao-Ping L., Zheng H., Wilder-Smith E. P. V. (2007). A feature selection method for multilevel mental fatigue EEG classification. *IEEE transactions on bio-medical engineering*.

[8] Zhao-peng L. I., Liu Y. (2007). Causation and countermeasure of the speed skating athete’s mental fatigue. *China Winter Sports*.

[9] May J. F., Baldwin C. L. (2009). Driver fatigue: the importance of identifying causal factors of fatigue when considering detection and countermeasure technologies. *Transportation Research Part F Traffic Psychology & Behaviour*.

[10] Elisabeth H., Lars R. N. C. (2004). Altered neuronal-glial signaling in glutamatergic transmission as a unifying mechanism in chronic pain and mental fatigue. *Neurochemical Research*.

[11] Zhang L., Zhang C., Feng H. E. (2015). Research progress on the interaction effects and its neural mechanisms between physical fatigue and mental fatigue. *Journal of Biomedical Engineering*.

[12] Moore T. M., Key A. P., Thelen A., Bwy H. (2017). Neural mechanisms of mental fatigue elicited by sustained auditory processing. *Neuropsychologia*.

[13] Lei Z., Wang Z., Wang X., Qi Y., Liu Q., Zhang G. (2016). Human fatigue expression recognition through image-based dynamic multi-information and bimodal deep learning. *Journal of Electronic Imaging*.

[14] Zhong Y., Zhang J. (2018). Task-generic mental fatigue recognition based on neurophysiological signals and dynamical deep extreme learning machine. *Neurocomputing*.

[15] Jap B. T., Lal S., Fischer P., Bekiaris E. (2009). Using EEG spectral components to assess algorithms for detecting fatigue. *Expert Systems with Applications An International Journal*.

[16] Arne D., Riam K. (2010). A review of EEG, ERP, and neuroimaging studies of creativity and insight. *Psychological Bulletin*.

[17] Lal S. K. L., Craig A. (2001). Electroencephalography activity associated with driver fatigue: implications for a fatigue countermeasure device. *Journal of Psychophysiology*.

[18] Ko L. W., Komarov O., Hairston W. D., Jung T. P., Lin C. T. (2017). Sustained attention in real classroom settings: an EEG study. *Frontiers in Human Neuroscience*.

[19] Talukdar U., Hazarika S. M., Basu A., Das S., Horain P., Bhattacharya S. Estimation of mental fatigue during EEG based motor imagery. *Intelligent Human Computer Interaction. IHCI 2016*.

[20] Chen C., Kun L. I., Qiuyi W. U., Wang H., Qian Z., Sudlow G. (2013). EEG-based detection and evaluation of fatigue caused by watching 3DTV. *Displays*.

[21] Iampetch S., Punsawad Y., Wongsawat Y. EEG-based mental fatigue prediction for driving application.

[22] Eldawlatly S., Zhou Y., Jin R., Oweiss K. (2008). Inferring neuronal functional connectivity using dynamic Bayesian networks. *BMC Neuroscience*.

[23] Ma M., Li Y., Xu Z., Tang Y., Wang J. Small-world network organization of functional connectivity of EEG gamma oscillation during emotion-related processing.

[24] Hata M., Kazui H., Tanaka T. (2016). Functional connectivity assessed by resting state EEG correlates with cognitive decline of Alzheimer’s disease–an eLORETA study. *Clinical Neurophysiology*.

[25] Zhang X., Li J., Liu Y. (2017). Design of a fatigue detection system for high-speed trains based on driver vigilance using a wireless wearable EEG. *Sensors*.

[26] Petroff O. A., Spencer D. D., Goncharova I. I., Zaveri H. P. (2016). A comparison of the power spectral density of scalp EEG and subjacent electrocorticograms. *Clinical Neurophysiology*.

[27] Murata A., Uetake A. Evaluation of mental fatigue in human-computer interaction-analysis using feature parameters extracted from event-related potential.

[28] Valer J., Daisuke T., Ippeita D. (2007). 10/20, 10/10, and 10/5 systems revisited: Their validity as relative head- surface-based positioning systems. *NeuroImage*.

[29] Li J., Struzik Z., Zhang L., Cichocki A. (2015). Feature learning from incomplete EEG with denoising autoencoder. *Neurocomputing*.

[30] Anat P., Libi S., Shlomo B. (2011). Motor and attentional mechanisms involved in social interaction--evidence from mu and alpha EEG suppression. *NeuroImage*.

[31] Seth A. K. (2010). A MATLAB toolbox for Granger causal connectivity analysis. *Journal of Neuroscience Methods*.

[32] Boucugnani L. L., Jones R. W. (1989). Behaviors analogous to frontal lobe dysfunction in children with attention deficit hyperactivity disorder. *Archives of Clinical Neuropsychology*.

[33] Stuss D. T. (2006). Frontal lobes and attention: processes and networks, fractionation and integration. *Journal of the International Neuropsychological Society Jins*.

[34] Dimitrakopoulos G. N., Kakkos I., Dai Z. (2018). Functional connectivity analysis of mental fatigue reveals different network topological alterations between driving and vigilance tasks. *IEEE Transactions on Neural Systems & Rehabilitation Engineering A Publication of the IEEE Engineering in Medicine & Biology Society*.

[35] Kar S., Routray A. (2013). Effect of sleep deprivation on functional connectivity of EEG channels. *IEEE Transactions on Systems Man & Cybernetics Systems*.

[36] Chee M. W. L. (2015). Limitations on visual information processing in the sleep-deprived brain and their underlying mechanisms. *Current Opinion in Behavioral Sciences*.

